# Online Morphological Adaptation for Tactile Sensing Augmentation

**DOI:** 10.3389/frobt.2021.665030

**Published:** 2021-07-20

**Authors:** Josie Hughes, Luca Scimeca, Perla Maiolino, Fumiya Iida

**Affiliations:** ^1^ Bio Inspired Robotics Laboratory, Department of Engineering, University of Cambridge, Cambridge, United Kingdom; ^2^ Oxford Robotics Institute, University of Oxford, Oxford, United Kingdom

**Keywords:** perception, tactile sensors, sensor morphology, object classification, morphological computation

## Abstract

Sensor morphology and structure has the ability to significantly aid and improve tactile sensing capabilities, through mechanisms such as improved sensitivity or morphological computation. However, different tactile tasks require different morphologies posing a challenge as to how to best design sensors, and also how to enable sensor morphology to be varied. We introduce a jamming filter which, when placed over a tactile sensor, allows the filter to be shaped and molded online, thus varying the sensor structure. We demonstrate how this is beneficial for sensory tasks analyzing how the change in sensor structure varies the information that is gained using the sensor. Moreover, we show that appropriate morphology can significantly influence discrimination, and observe how the selection of an appropriate filter can increase the object classification accuracy when using standard classifiers by up to 28%.

## 1 Introduction

Morphology and structure has been shown to significantly alter and affect tactile information (Scimeca et al., 2018); Hughes and Iida, 2017). The optimization of sensor morphology can therefore significantly aid our ability to understand the world and perform complex sensory tasks. In biological systems such as the human hand, we see complex sensor morphologies that have evolved across both many generations and through the developmental process of an individual. This results in sensory receptors with different structures, with spatial distribution, mechanical properties and interfaces that are optimized for performing tactile tasks (Maeno et al., 1998); Vallbo and Johansson, 1984. However, it remains challenging to develop artificial sensing technologies that allow for considerable variation and evolution in morphology and structure, and to develop computational design approaches for optimizing or designing the structure of the sensor (Iida and Nurzaman, 2016). In particular, there remains a key open challenge of how a sensory system can be designed, or adapted online for an unseen or continuously adapting tactile sensing task.

The goal of this research is develop a mechanism to adapt or alter the sensor morphology online, to allow for improved tactile discrimination between objects. In addition, to do this without sacrificing loss of sensory resolution or capabilities, while still producing a significant range of morphological adaption to allow appropriate compensation and adaption for the discrimination task. The key performance indicator of this work is to demonstrate increased object discriminative performance when varying sensor morphology online.

Previously, research has focused on the optimization of sensor layout exploring how sensor structure can be optimized to maximize information gain (Thuruthel et al., 2020), to allow the morphology to perform localized processing (Hughes and Iida, 2017), to amplify or improve the sensitivity of the response (Fend et al., 2004), and to optimized for a specific task (Qi et al., 2019). In addition, the relationship between sensor morphology and action for perception has been explored (Huang et al., 2019; Scimeca et al., 2020b; Scimeca et al., 2021). These approaches highlight the importance of sensor structure and morphology when performing action based perception tasks (Bernth et al., 2018). This research direction highlights some of the trade-offs that existing in optimizing sensor morphology. For example, introducing softer sensors offers increased compliance (Margheri et al. (2012); Brown et al. (2010); Pfeifer et al. (2014); Hinitt et al. (2015)) allowing the haptic or sensory devices to conform to the surface of the object itself, increasing the contact area (Scimeca et al., 2018; Hughes and Iida, 2018). The use of wrinkled surfaces has also been shown to be useful for increased perception in tactile sensors which combined with motion (Ho et al., 2017; Trinh et al., 2019; Qi and Ho, 2020). However, the use of elastic materials can form some mechanical “low-pass filtering of the stimuli, with the potential to affect the spatial and force resolution of the sensor (Shimojo, 1997; Scimeca et al., 2018). Thus, although sensor morphology can aid sensing capabilities for certain tasks, it is not necessarily generalizable to a wide variety of tasks. Having online control of morphology would allow the benefits to be achieved in a far more generalize way. However, from a technology perspective this is challenging. There have been only limited demonstrations of sensors which allow for adaptation. Examples include *in situ* adjustable sensor morphology utilizing hot melt adhesive (Nurzaman et al., 2013), or tuning the sensitivity and dynamic range of sensors through material properties of liquid sensors (Liao et al., 2015).

We propose a mechanism for altering the sensor response online by using an morphologically adaptive filter which sits on the surface of a tactile sensor forming a physical interface layer between the tactile sensor and the environment. The filter is constructed from jamming particles (Brown et al., 2010) encased with a soft outer structure and connected to a vacuum pump with allows the stiffness of the filter to be altered online. The stiffness change properties can be utilized by allowing the filter to be molded to a desired shape when low stiffness by using molds or templates. The filter can then be stiffened allowing the shape to held, providing both online morphological adaption and allowing the sensor properties to be maintained. We propose that this allows for improved sensing as this morphological adaption allows the surface shape to be adapted and shaped such that it better fits the test object increasing the contact area and increasing the information content of the response. Secondly, the filter morphologies can be set to physically restructure the sensory information to allow improved sensory discrimination between otherwise similar objects.

In this paper we demonstrate the advantages of the morphologically adaptive filter on a set of test objects which exhibit different features (stiffness, edges, texture) to explore the improvements offered by the filter in discriminating these different feature types, by changing sensor morphology. As a demonstration, we then test the approach on a real object set. We show the improvement in classification that can be achieved with this method, however, after this preliminary introduction of the filter there is much scope to explore how to optimize the sensor morphology or any unseen tasks or sensory task. We show that the object discriminative performance can be increased by 28% when using the adaptive filter in comparison to an non-adaptive approach.

In the remainder of this paper we introduce the filter structure in Section 2 alongside the data-processing and learning methods. The specific implementation and experimental details are given in Section 3 followed by the experimental results in Section 4. The final section provides and discussion and conclusion exploring the advantages offered by this approach and direction for further exploration of this approach.

## 2 Methods

### 2.1 Jamming Morphological Filter

**FIGURE 1:**
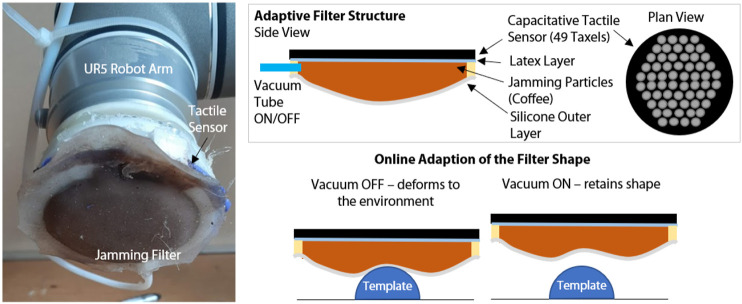
The morphologically adaptive filter fitted to the sensor mounted on a UR5 robot arm, showing the structure of the filter and the mechanism by which the filter can change shape online.

The jamming based morphological filter is placed over a circular capacitive sensor disk which has 50 “taxels” providing high sensitivity and spatial distribution over the surface of the sensor. The sensor provides measurement with a resolution of 16 bits corresponding to a variation of capacitance proportional to the pressure acting on top of the sensor. Details of the specific sensor and its fabrication have been previous reported (Maiolino et al., 2013; Scimeca et al., 2018). The jamming filter is constructed from a thin flat latex layer, a plastic ring and an deformable silicone skin, which have been sealed to create an airtight system. This is filled with jamming particles, coffee, with a tube connected which allows a vacuum to be applied. This enables the stiffness of the filter to be varied online, transforming it from a low stiffness deformable filter, to a high stiffness, hard filter (Figure 1).

To demonstrate the advantages that can be achieved with this jamming filter we perform some simulated experiments using FEA. To do this, we explore the pressure patterns which are exerted on the surface of the capacitive sensors when different modalities of adaption of the sensor are performed. This is performed using FEA to first determine the shape of the sensors when it is unjammed by modelling the filter as a elastomeric material being brought into contact with the object with a fixed displacement (3 mm). From this process we extract the mesh, or resultant morphology of the filter from the simulation. To then simulate the sensor response, we take this mesh filter shape, and change the material stiffness to represent the jammed filter - using the Young’s Modulus of a hard plastic material (50 MPa). We then obtain a representative sensor response by visualizing the pressure distribution of the back side of the filter when the jammed filter in brought into into contact with an object with a 1N distributed load. This is equivalent to the conditions in which the filter is used on the UR5 robot arm.

**FIGURE 2:**
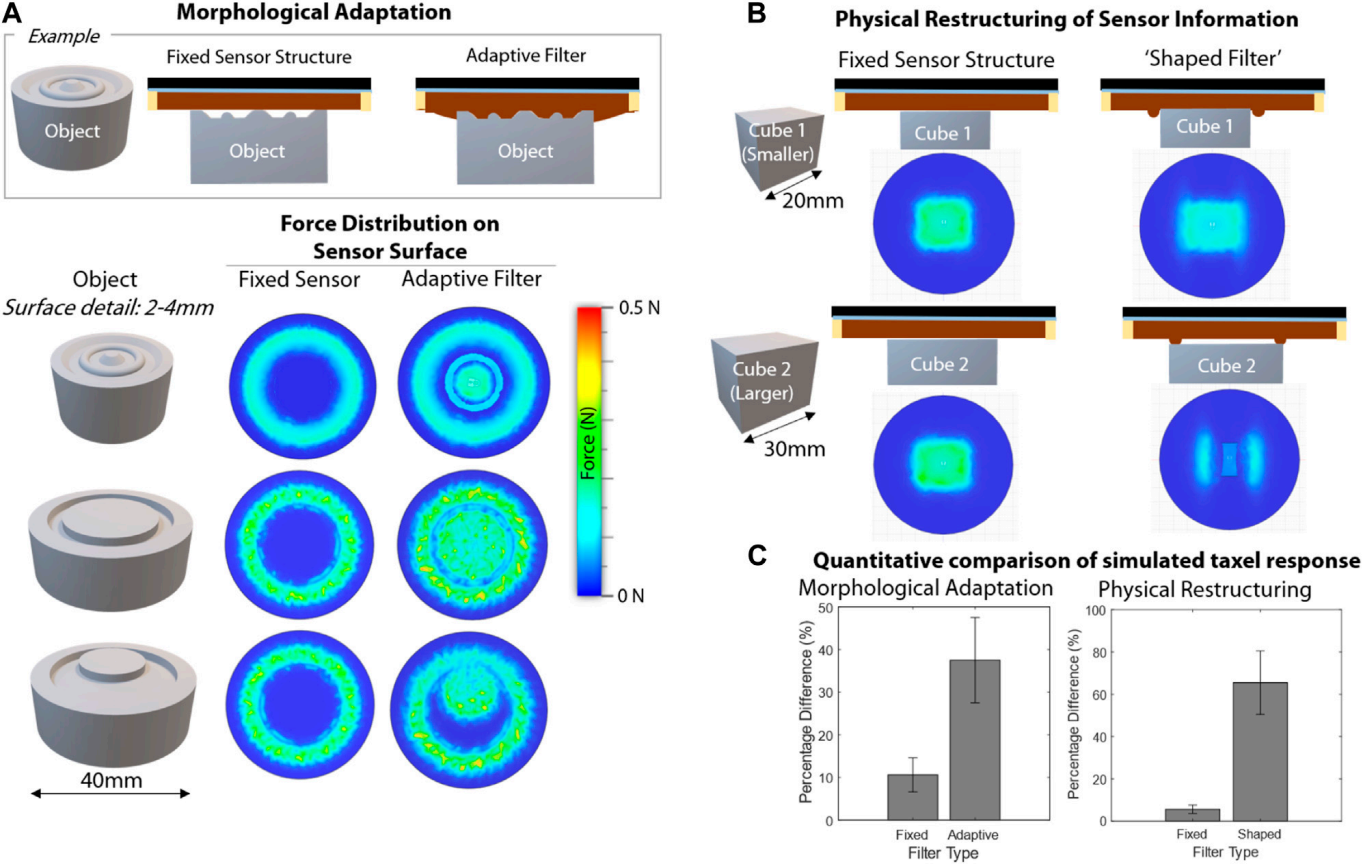
Illustration of the advantages offered by the adaptive filter showing FEA based simulations of the force profile on the surface of the sensor for **(A)** morphological adaptation showing a simulation of the force on the sensor surface for the fixed and adaptive filters for various objects with similar structure, but varying surface detail, **(B)** Physical reconstructing of the sensor information for cubes of different sizes, whe used a flat fixed sensor, or a shaped filter **(C)** Quantative comparison of the simluated responses, left - the percentage of differences in pixesls for the FEA responses shown in **(A)**, and right, the difference for thoose shown in **(B)**.

Using this FEA analysis we can demonstrate two mechanisms by which this stiffness change offers key advantages. Firstly, it offers a compromise between soft and rigid sensors. It provides compliance and physical adaptation as seen in soft sensors, increase the contact area between the filter and the surface. However, when jammed, we do not see some of the more negative reduction in spatial and force resolution that can be seen in with soft sensors. As shown in Figure 2A we explore this by simulating the response from the fixed and adaptive filter for similar objects but with different, lower surface detail. In all cases we see that the Adaptive Filter leads to force transmission between the object and filter for the surface detail, so more surface information is captured. With the rigid sensor, the filter structure is not in contact with the surface detail of the object so gets no response from this detailing on the object. We also show these results quantitatively in Figure 2C left, were we consider the percentage difference in pixels of the FEA images for these different objects when seeing the fixed and adaptive filters. These results show that when used the fixed sensor, the objects have a low difference, appearing to be similar, however, the adaptive filter leads to far greater percentage differences in the responses, aiding classification or detection.

The second advantages of the filter is that it is possible to change the morphology of the filter online. This is achieved by “shaping” the filter when unjammed by forming the filter around a template and then jamming the filter to hold on to the shape. The morphology or structure of the filter can thus be chosen to aid in the task by inducing desired differences in the sensor response. For example, to offer improved discrimination between two objects that otherwise may appear similar to the sensor. Take two cubes, one slightly bigger than the other, which induce a very similar sensor response. By shaping the filter, we can introduce morphology into the filter which results in the two objects leading to a significantly different response when tested. This is highlighted in Figure 2B where example force profiles are demonstrated for a fixed sensor structure and a shaped filter. In Figure 2C right we also show these results quantitatively, by again considering the difference between the pixels of the FEA analysis. We see for the fixed filter the images have a low difference, however, for the shaped filter the difference is much higher, at over 60%.

In summary, changing the shape of the filter can induce the sensor response to be significantly different for the two objects in comparison to using only a fixed sensor structure. This could be viewed as the filter providing a physical memory, which is then amplified when it stiffens through the process of jamming.

The design of the template objects should seek to utilize these mechanisms to allow for optimal discrimination between objects. In this work we consider a small set of filters to demonstrate and explore the effectiveness of this approach. To explore the morphological adaptation we have a fully soft, hard flat and hard ridged filters. In addition, we have an “adaptive filter” where the template molds are the objects being tested themselves. This should aid discrimination as should act like a key and lock mechanism, where the adaptive filter for a specific object should “fit” best for the object on which it was made. These filter have been selected to highlight and explore the different mechanisms and advantages of this sensor filter, as indicated above.

**FIGURE 3:**
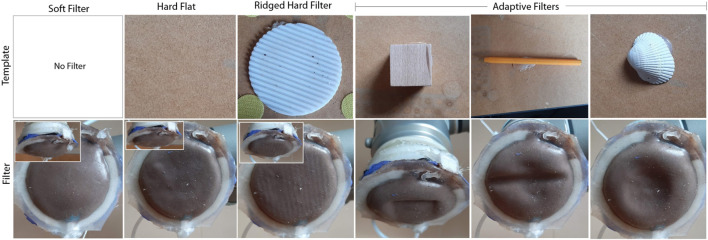
Examples of template structures and the corresponding deformation pattern of the filter when the online adaption of the filter is performed.

To demonstrate the physical adaptation that can be achieved using the adaptive filter Figure 3 shows the deformed filter for various template objects. As shown in Figure 3 the filter takes on the shape of the surroundings, allowing for the texture and 3D shape of the filter to be adapted online.

### 2.2 Control

We perform controlled experiments with a 6-DoF UR5 industrial robot arm. We manually teach the robot the location of the objects to palpate, and set the starting position of each experiment with the robot’s end-effector aligned normally to the surface of the object to touch. We control the arm in Cartesian coordinates at a approximately 125 Hz per second. Each experiment consists of two phases, i.e., sensor molding, and object touch.

The object molding phase is used to automatically induce a particular morphology onto the surface of the sensor filter. The filter is adapted online by pressing the sensor filter’s surface into the template object, to deform the filter, after which a vacuum is applied to the filter, activating the jamming mechanism, and the “memory” shape of the mold is maintained onto the filter’s surface.

**FIGURE 4:**
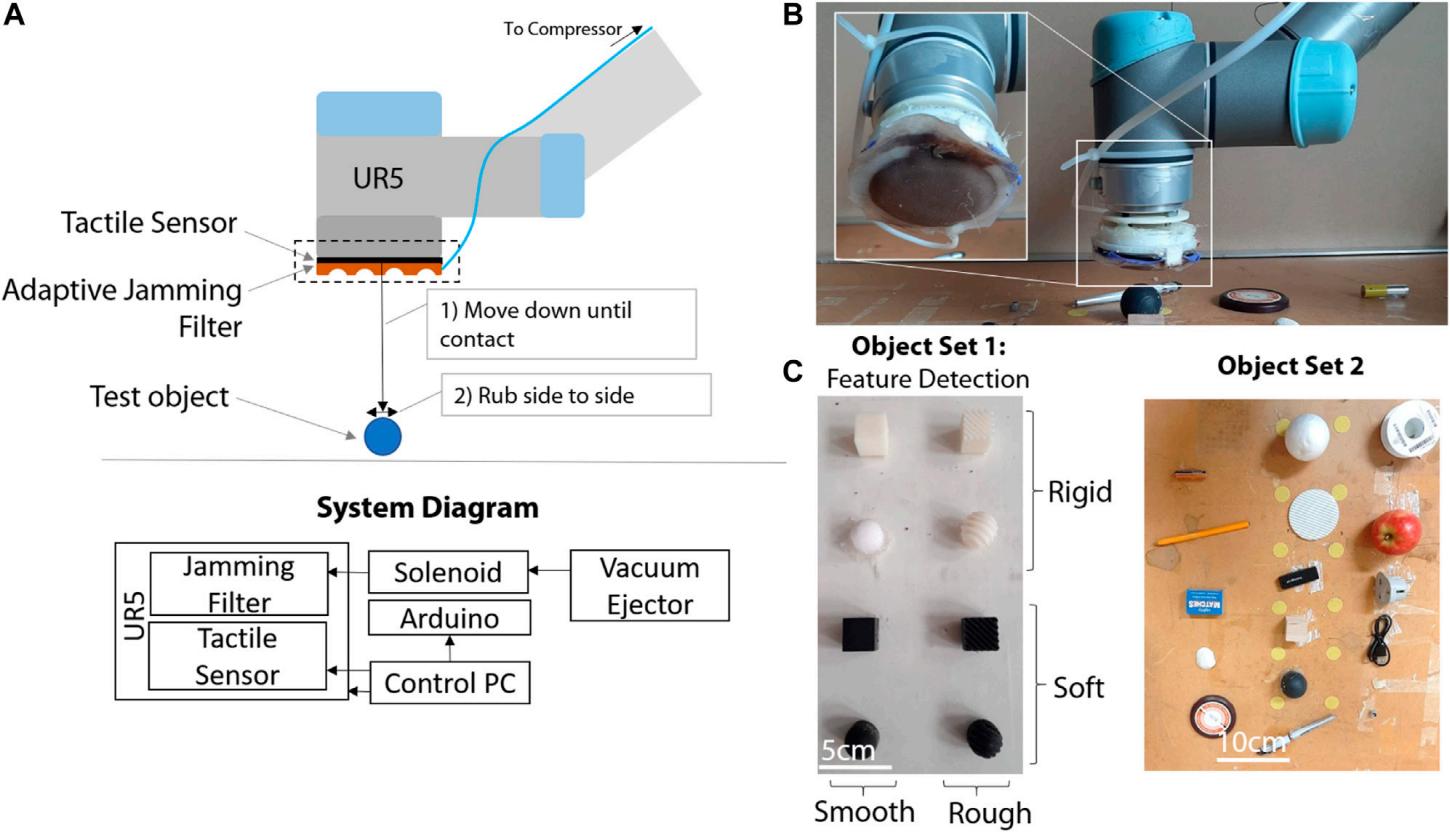
**(A)** Diagram showing the components of the experimental setup and a block diagram of the system, **(B)** Experimental setup showing the adaptive filter, the UR5, **(C)** Test object sets. The “soft” objects are printed in Tango Black (26–28 Shore A hardness) using a Objet Connex 3D printer, and the rigid objects with VeroWhite (83–86 Shore D Hardness). The rough objects have ridges of 2.5 mm separation.

The object touch phase consists of a controlled 5-s interaction between the sensorized end-effector and the object under touch. For each object to touch, the end-effector is controlled to move normally downward until a touch event is detected by the capacitive tactile sensor at its extremity. The touch event consists of a raise, in any of the 50 taxels, by more than 5% of their reading range. After contact is detected, the robot proceeds to perform a “rubbing motion”, by moving diagonally in the x-y plane of motion (Figure 4) for 5 s. From the detection of touch to the end of the 5-s motion, the tactile sensor is sampled at 50Hz. We thus retrieve a total of 250 tactile images for each experiment, each containing responses from 50 different taxels. This brings the dimensionality of each tactile experiment to a 12,500 dimensional vector.

### 2.3 Clustering and Information Measures

The following section details the data processing and handling techniques which are used to analyze and reduce the sensory information gained during experiments.

#### 2.3.1 Dimensionality Reduction

We perform several sets of analysis to assess the quality of the tactile information from the different filters. First, we assess the capacity to perform dimensionality reduction on the data. We choose Principal Component Analysis (PCA) to perform dimensionality reduction. Inherently we thus assume linearity in the dimensions of the tactile data.

Let **W** be a matrix, where each row corresponds a tactile image sequence from each touch experiment. The sequence is a one-dimensional concatenation several sensor readings over the same touch experiment. The number of rows in **W** corresponds to the number of touch experiments performed.

We use PCA to reduce the dimensionality of W, where $p_{i}$ is its $i^{th}$ principal component (Abdi and Williams, 2010). We cal W, the projected matrix.

After PCA, we analyze the amount of information retained by each principal component. Note that by the earlier underlying assumptions we will refer to “retained information” as the proportion of variance in the data captured by each of the new axis, as defined by PCA.

#### 2.3.2 Cluster Analysis

We analyze the tactile data by the amount of “structure” present within, after probing each object several times. One measure of structure which can be used in this scenario is the Silhouette score (Rousseeuw, 1987). The Silhouette is a measure which considers the trade-off between the intra-cluster distance of sensor values for objects belonging to the same class, and the nearest-cluster distance between clusters of objects belonging to different classes. This measure has previously been successfully used for the analysis of tactile information in discrimination tasks (Scimeca et al., 2020a).

The silhouette score s(i) for cluster *i* can be computed as:

$$s\left(i\right)=\frac{b\left(i\right)-a\left(i\right)}{\text{max}\left(a\left(i\right),\;b\left(i\right)\right)}$$ (1)

where a(i) is the mean intra-cluster distance of cluster *i*, and b(i) is its mean nearest-cluster distance. We will refer to the silhouette score *s* as the average score for each cluster in the data, i.e., :

$$s=\frac{\sum_{i=1}^{K}s\left(i\right)}{K}$$ (2)

The score will thus be a number s∈[−1,1], where data exhibiting more structure will score higher *s* values. In turn, higher *s* values will signify more “separable” cluster of objects in sensor space, which may in turn lead to better classification performance.

### 2.4 Learning/Classification

We perform further experiments on the quality of the tactile information by training and comparing several machine learning algorithms on the data retrieved by the various filter modes. We pick 9 standard machine learning classifiers to perform this classification: Nearest Neighbors (Goldberger et al., 2004), Linear SVM (Mayoraz and Alpaydin, 1999), RBF SVM (Yan et al., 2006), Gaussian Process (Williams and Rasmussen, 2006), Decision Tree (Steinberg and Colla, 2009), Random Forest (Cutler et al., 2012), Feed Forward Neural Network (Rumelhart et al., 1986), Naive Baise (Schütze et al., 2008) and QDA (Hootman, 1992). All the models were implemented with the Scikit-learn library (Pedregosa et al., 2011). Given the purpose of this comparison, we fit each model with its default hyper-parameters from (Pedregosa et al., 2011), and do not perform any particular hyper-parameter tuning. To compare the results, we randomly select 70% of the lower-dimensional PCA projections of each tactile image data, and leave the remaining 30% for testing. This is performed upstream, thus all models will be compared on the same data splits. As no ad-hoc hyper-parameter fitting procedure was performed in each classifier, it was not necessary to select separate data-points for validation.

We explore a number of different classifiers to allow us to observe how each algorithm performs when given the data generated by each filter. Here we hypothesize that “higher quality” data should allow several standard algorithms to accurate classify each object under touch, while “low quality” data would make object discrimination harder, or impossible. This should be even more visible in lower dimensional projections.

## 3 Experimental Setup

To explore and understand robotic palpation, we use a 6 Degree of Freedom (DoF) UR5 Robotic Arm that can perform complex end-effector trajectories (Figure 4A). An adaptive jamming filter is attached onto the surface of a capacitive tactile sensor as previously described, and a template is used to shape the filter at will. The jamming of the filter is performed by applying a vacuum to filter, which is controlled via a solenoid triggered valve connected to a vacuum supply. The solenoid is controlled via an Arduino which communicates with the control PC over serial. This allow for rapid change of the stiffness properties online. We consider two main object tests sets (Figure 4C). Object set one contains similarly sized objects but with variation in features (shape, texture and “edginess”) to allow us to explore how the filters help identify different feature types. Object set two is a demonstration set of “real world” objects, to demonstrate how this approach can assist with classification of real world objects.

## 4 Results

### 4.1 Feature Based Discrimination Task

In these first set of experiments we consider a set of similar sided “test” objects which have some variation of feature (stiffness, roughness, edges), to explore how the adaptive filter can assist in identifying specific objects. The analysis was performed on a set of 640 touch experiments, where 4 filters were used to touch 8 objects from the top, and each touch experiments was repeated 20 times. Moreover, the tactile sensor is sampled at 50 Hz over 5 s, where each sample is a 50-dimensional array of taxel values, bringing the dimensionality of each of the 640 data-points to 12,500.

#### 4.1.1 Filter Responses

**FIGURE 5:**
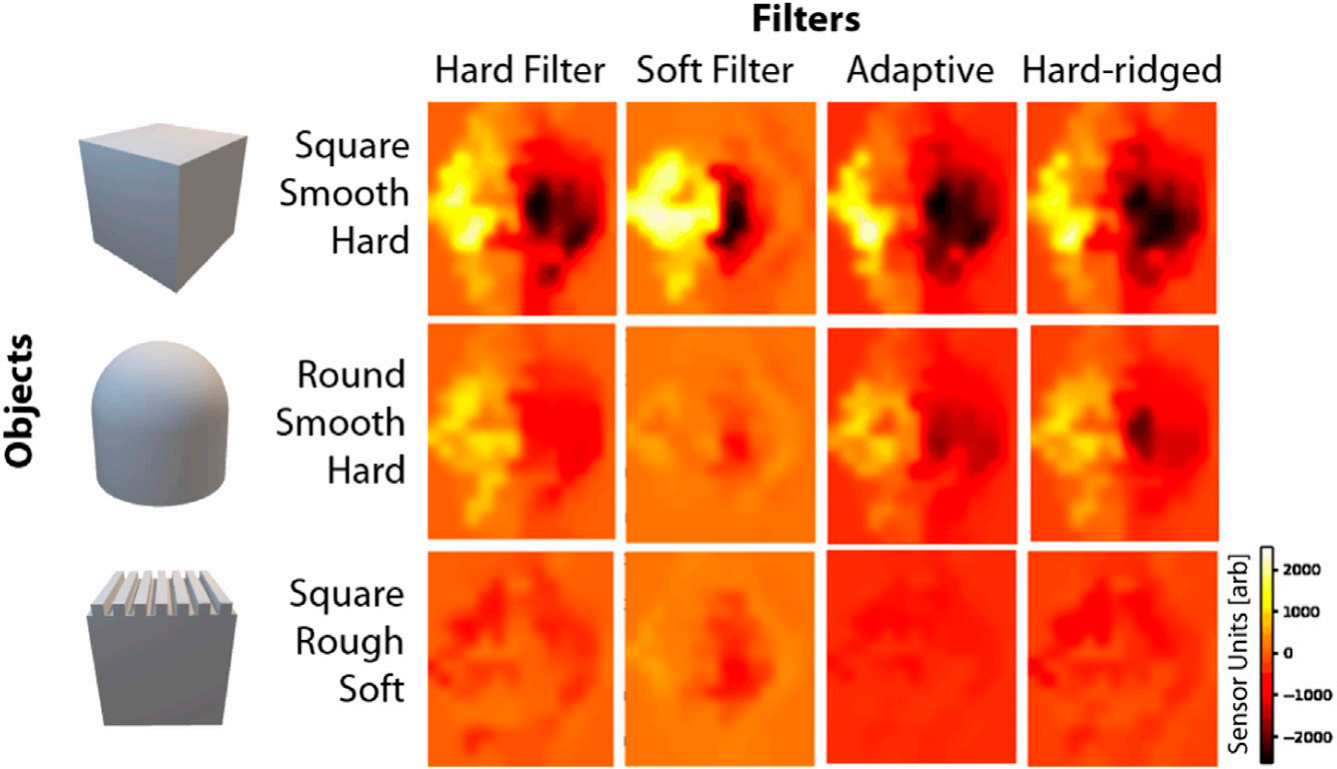
Raw tactile sensor activation after 2.5 s from the beginning of the touch experiment, when touching three objects with four different filter shapes. Brighter colors correspond to higher sensor values.

To demonstrate the ability of the adaptive filter to significantly influence the tactile response of the sensor upon touch, we show the raw tactile sensor response in Figure 5 for three different of objects being touched with different filters. The touch experiments are performed as explained in Section 2.2, with the tactile sensor activation pattern shown after 2.5 s from the start of the 5-s touch experiment (Figure 5). By comparing the images in each row in the figure, it is clear how the filter can influence the sensor response, and how this influence is different depending on the object under contact. It is important to consider that this figure just reports the sensor response at a single time step, and thus there is variation in the filters response across the entirety of each touch experiment.

**FIGURE 6:**
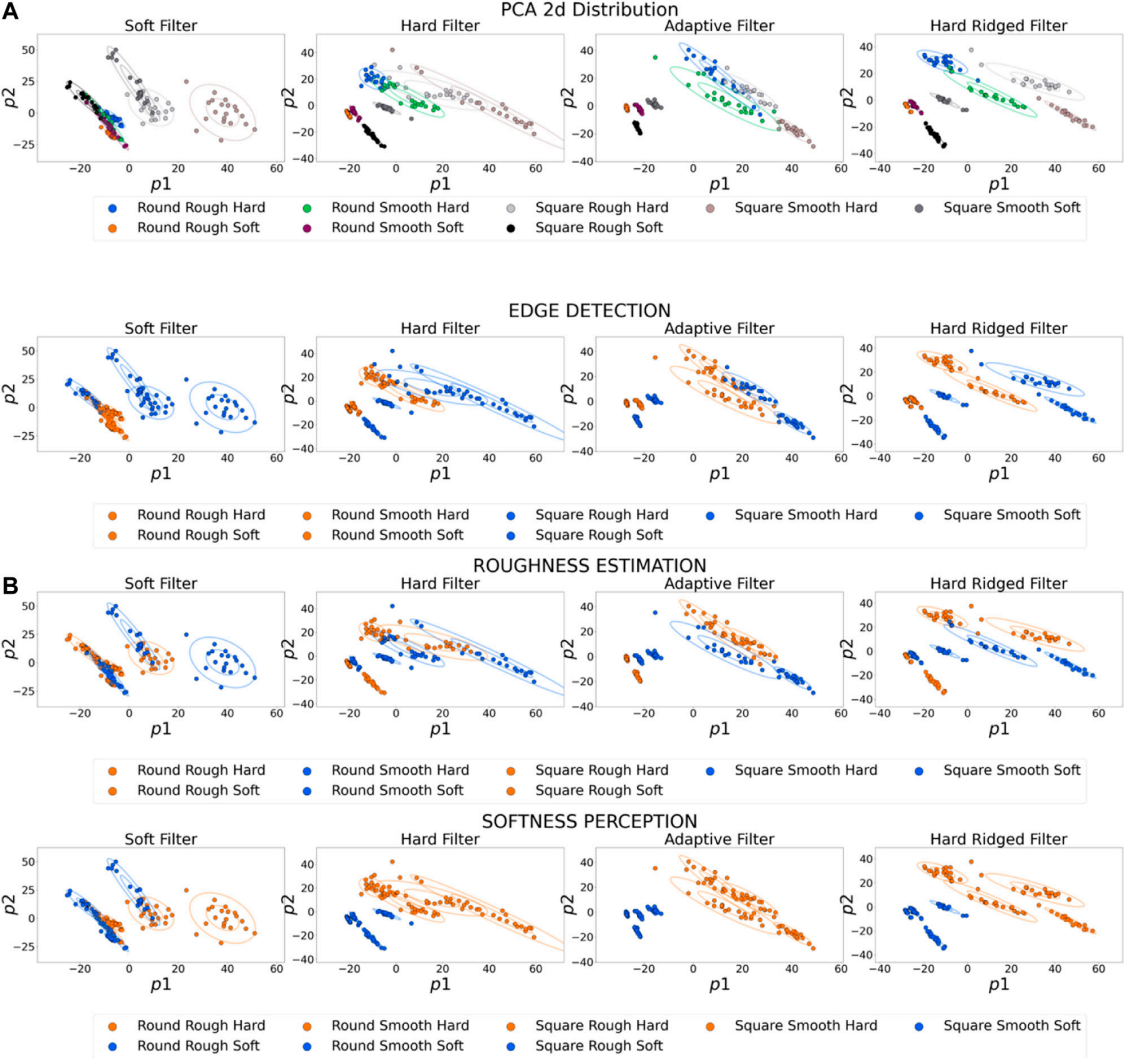
PCA 2D projections of the sensor response. Each figure is generated via touch experiments with a different filter shape.

In Figure 6 we show the distributions of the 2D PCA-projected tactile sensor response, for the different filter shapes. Here we refer to the filter as being “hard” when jammed, and being “soft” when un-jammed and the filter is free to deform. The figure shows a very diverse range of distributions depending on the filter utilized. The first important consideration lies with the ability of some filters to clearly separate the objects in sensor space. This is the case for the hard ridged filter (right-most plot in Figure 6A). Others, instead, show a more cluttered picture, where some objects are almost impossible to discriminate. This is the case, for example, the purely soft filter (left-most plot in Figure 6A). A second important consideration lies with the ability of some filters to optimize separation for particular tasks. Figures 6A–C shows the same distributions, but color-coded based on particular features, i.e., roughness, edges and softness. The Hard Ridged filter and the Adaptive Filter can achieve a good separation of objects in all three tasks. The Stiff filter can achieve good softness perception, but is understandably least apt at roughness estimation, and struggles edge detection. The Soft filter can achieve a measure of edge detection, and softness perception, but cannot show a clear separation for roughness estimation. These results results really highlight the need for online adaption and evolution and optimization for a specific task - no one filter is optimal for all tasks, and different filters have different relative advantages.

### 4.2 Performance and Analysis

**FIGURE 7:**
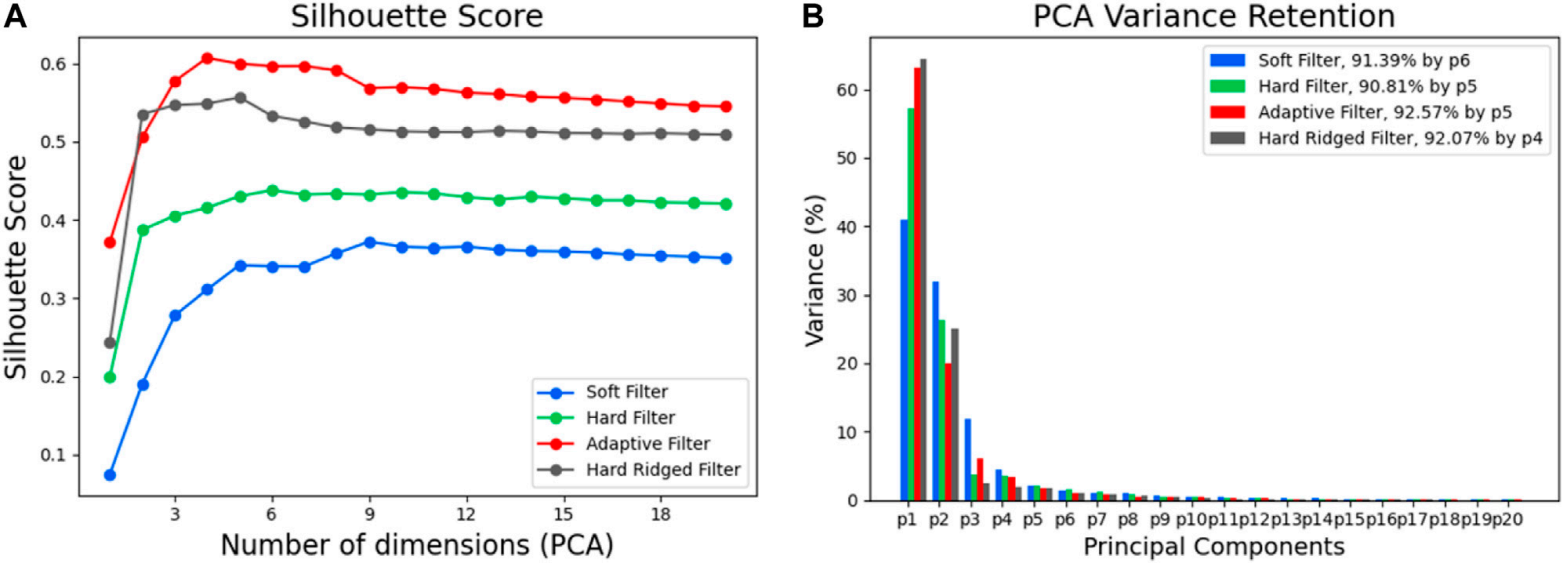
**(A)** The silhouette score for increasing number of dimensions for the different filters when classifying the different objects. **(B)** The variance in the principle components for the different filters.

To analytically investigate the results in Section 4.1.1, we can consider the Silhouette score; a measure of information structure directly dependent on the separation of object clusters in sensor space. We compute the Silhouette score across a varying number of PCA projected dimensions, so as not to show bias by our choice of dimensionality reduction. Figure 7A shows the Silhouette score of the sensor data as a function of the dimensionality reduction in PCA. First, we again notice that different filters provide a very different amounts of structure. As expected, both the Adaptive and Hard Ridged filters achieve the highest score across dimensions, with the Hard Ridged filter inducing more structure than the Adaptive Filter in two dimensions, but less so in all others.

This picture is also confirmed in Figure 7B, which depicts the explained variance for each principal component with varying filters. In the figure, we can see that by using the Hard ridged or Adaptive Filter we can retain over 60% of the variance with only one projected dimension, while we can explain over 90% of the total variance in the data with 4 or 5 dimensions in total.

**FIGURE 8:**
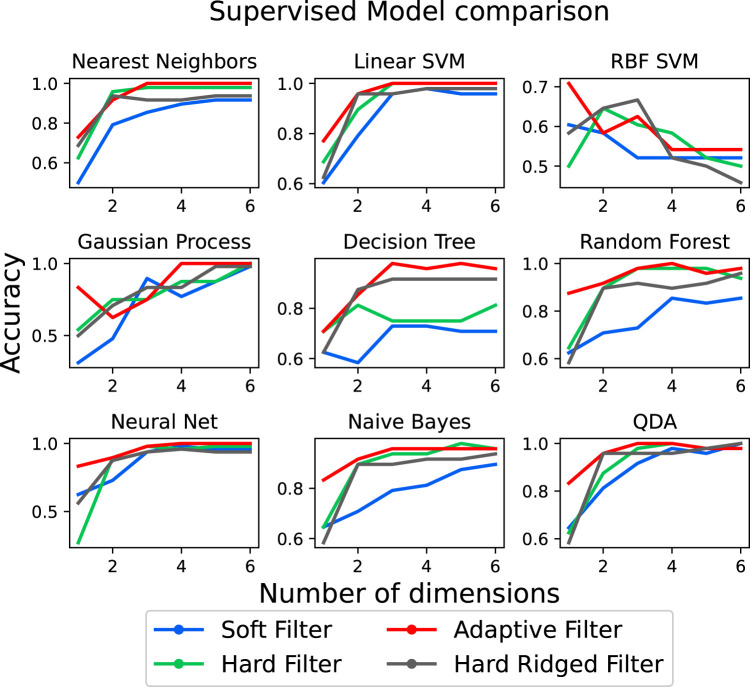
Test accuracy when performing classification of the different objects using the nine different classifiers as a function of the PCA dimensions.

Finally, we wish to verify if the observed influence of the filter on classification performance. We do this by comparing 9 standard classifiers with the data obtained by all filter shapes. The data is fit to each classifier as described in Section 2.4. Moreover, each classifier is trained separately with respect to the data generated by each type of filter, in turn. The random test and training splits for the data generated by a particular filter are kept consistent across all classifiers. Figure 8 shows the classification accuracy achieved with the data from each filter, as a function of the number of projected dimensions in PCA. As before, we do so to ensure there is no dependency between performance and dimensionality reduction.

We observe that the predictions loosely follow the estimations of information structure by the Silhouette score. The data generated by the Adaptive and the ridged filter can achieve state of the art results in 6 out of 9 classifiers, with the purely soft filter generally under-performing in all scenarios. It can be also noticed that the relative ordering loosely respects the silhouette predictions, where for a 2-dimensional projection the Stiff Ridged filters outperforms or equates the Adaptive filter with 8 out of 9 classifiers, while generally under-performing on other dimensions.

Table 1, illustrates the average accuracy performance of each of the 9 classifiers considered across the first 50 projected PCA dimensions. The first important consideration is that, on average the Adaptive Filter achieve the highest performance overall (99.49%) as well as the highest number of best performing classifiers overall (4/9). Moreover, depending on the classifier, it is possible to achieve up to a 24.37% increase in classification accuracy when considering an appropriate morphology. This validates the need of adaptive morphologies in classification tasks.

**TABLE 1:** Accuracy of 9 classifiers to discriminate across 8 objects. Each of the accuracies reported are averaged across 50 runs. Each run corresponds to the respective classifier fit on PCA projected tactile data from one up to 50 dimensions.

Classifier	Soft Filter	Hard Filter	Hard Ridged Filter	Adaptive Filter	% Increase
**Nearest Neighbors**	95.62	97.15	95.75	**99.28**	3.66
**Linear SVM**	98.43	99.15	98.94	**99.45**	1.02
**RBF SVM**	42.01	**44.09**	39.97	43.96	4.12
**Gaussian Process**	79.42	97.53	**97.62**	96.60	18.2
**Decision Tree**	72.02	81.68	90.73	**96.39**	**24.37**
**Random Forest**	78.66	**84.57**	81.55	80.61	5.91
**Neural Net**	98.30	98.09	98.00	**99.40**	1.4
**Naive Bayes**	**93.71**	92.43	93.03	92.22	1.49
**QDA**	82.78	89.12	**91.96**	87.67	9.18
**Best Performance Count**	1	2	2	**4**	

### 4.3 Demonstration Object Discrimination Task

To demonstrate the generality of our approach, we perform test experiments on a set of 17 objects, which vary in shape, dimension, softness, texture and more (Figure 4C, Object Set 2). We pick the highest performing filters from previous experiments: the stiff Filter, the Hard Ridged Filter and the Adaptive Filter. The analysis was performed on a set of 510 touch experiments, where 3 filters were used to touch 17 objects from the top, and each touch experiments was repeated 10 times. Moreover, like before, each experiment is a 12,500 dimensional array of taxel values over a 5 s period.

#### 4.3.1 Sensor Space Distribution

**FIGURE 9:**
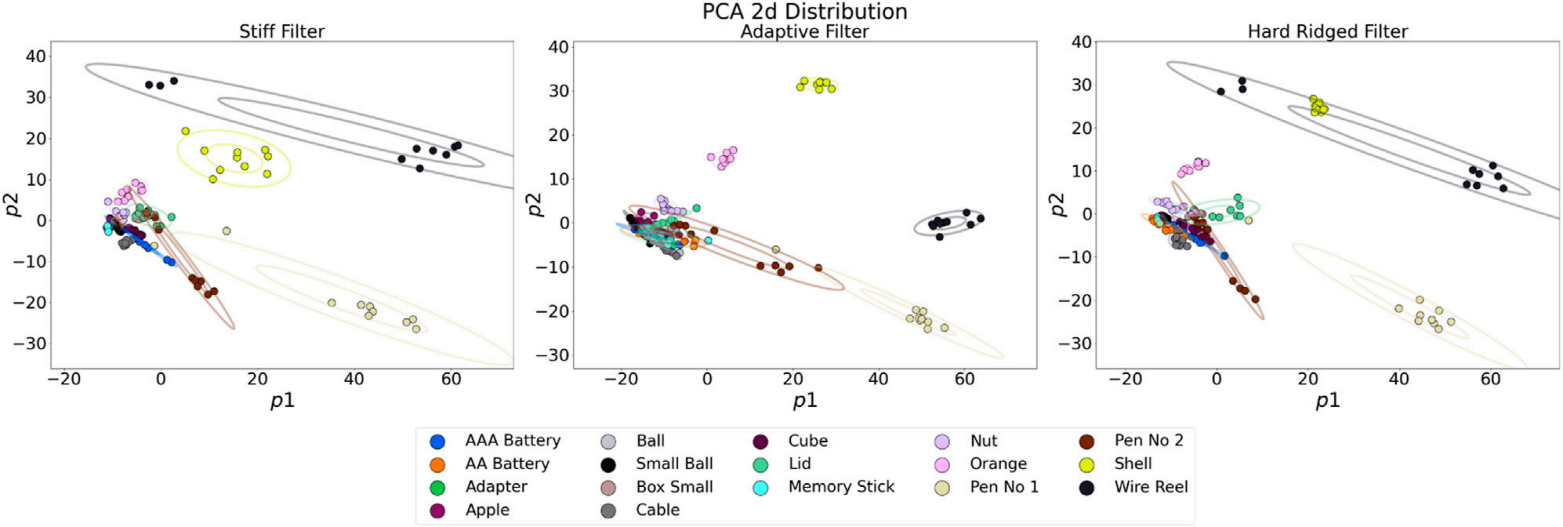
2D projections of the PCA analysis of the sensor response for the experiments when performed with the three different filters (stiff, adaptive, and hard ridged).

Figure 9 shows the PCA 2D projections of the sensor response during the experiments with the set of 17 general objects. The first observation we can make is that as expected, the distribution of the 2D projected sensor response changes depending on the filter used. Moreover different morphologies allows for the better discrimination of different objects.

The Adaptive Filter induces object clusters with less variability. This, in turn, allows for the better differentiation of the shell, the wire reel, the orange and Pen No 1. It is also possible to distinguish the nut and a second pen, however all other objects present very similar distributions. The Hard Filter present sparser distributions, which can nonetheless separate the wire reel, the shell, the two pens and the orange. Although by a smaller margin, it is also possible to identify clusters for several other objects including the memory stick, the nut, the cable, and the AAA battery. The Hard Ridged Filter induces a clear 2D separation of the shell, the wire reel, the orange, Pen No 1, the nut and the lid.

**FIGURE 10:**
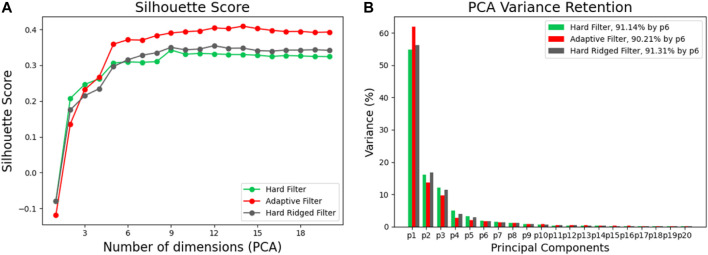
**(A)** The Silhouette score for the different filters for increasing numbers of dimensions. **(B)** The variance in PCA for the principle components for the three different filters.

It is also important to consider the separation in sensor space for the higher dimensions, by observing the Silhouette score across different dimensions (Figure 10A). Here the information structure score for the data generated by the Adaptive filter exceeds the other two counterparts only for projections including 5 or more principal components. In Figure 10B we observe how the explained variance for each PCA dimension is also affected by the filter shape. As previously, the adaptive filter can still explain more than 60% of the variance in the first dimension, while all filter retain a little over 90% of the variance across only after considering at least 6 PCA projected dimensions.

**FIGURE 11:**
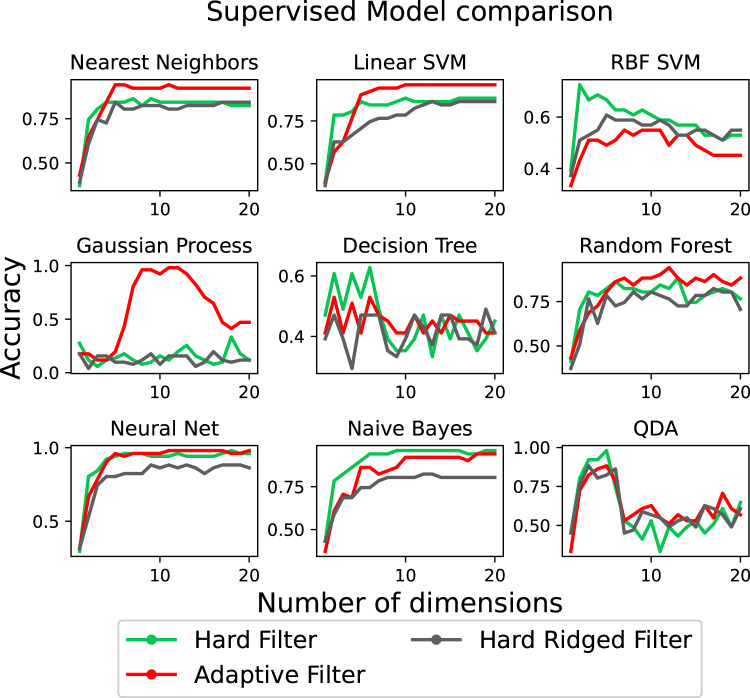
The accuracy when classifying the objects using nine different models when using the three different filters for increasing number of PCA dimensions.

Finally, we observe the ability of the previously considered 9 classifiers to distinguish among the 17 touched objects. We perform this across varying numbers of PCA projected dimensions, and report the findings in Figure 11. In the figure we can see how the Adaptive Filter achieves state of the art performance with 6/9 classifiers, the Stiff Filter with 5/9 classifiers and the Ridged Filter with 1/9 classifiers. As clear from the image, the choice of dimensionality is also an important factor to consider.

#### 4.3.2 Accuracy

Table 2, instead, illustrates the average accuracy performance of each of the 9 classifiers considered across the first 50 projected PCA dimensions. The table highlights two important facts, one, the adaptive filter is capable on average to achieve the highest accuracy score 6 out of 9 times; and two, depending on the classifier, it is possible to achieve up to a 28.37% increase in classification accuracy when considering an appropriate morphology.

**TABLE 2:** Accuracy of 9 classifiers to discriminate across 17 objects. Each of the accuracies reported are averaged across 50 runs. Each run corresponds to the respective classifier fit on PCA projected tactile data from 1 up to 50 dimensions.

Classifier	Hard Filter	Hard Ridged Filter	Adaptive Filter	% Increase
**Nearest Neighbors**	82.99	81.63	**91.24**	9.61
**Linear SVM**	88.00	82.43	**92.72**	10.29
**RBF SVM**	51.66	**51.82**	45.98	5.84
**Gaussian Process**	19.77	15.41	**43.78**	**28.37**
**Decision Tree**	42.90	41.26	**42.94**	1.68
**Random Forest**	71.67	69.43	**75.27**	5.84
**Neural Net**	**94.92**	86.15	94.56	8.77
**Naive Bayes**	**93.92**	81.39	89.84	12.53
**QDA**	56.18	59.18	**61.70**	5.52
**Best Performance Count**	2	1	**6**	

## 5 Discussion

In this work we introduced a jamming based sensory filter that allows for the structure and properties of the sensor to be varied online. Using this filter we demonstrate how using template objects, and test objects, the morphology can be varied online which provides significant change to the sensor response, with some filters enabling improved separation or classification of the objects in sensor space. By choosing the best of the four filter techniques we introduce, classification of the feature based object set can be improved by 24.37%. Particularly notable, for the real world object data set, it was possible to achieve up to a 28.37% increase in classification accuracy when adapting the filter online with the appropriate morphology.

An important related state remark following the performance of the 9 different classifiers used in this work, is the importance of data “pre-processing” by physical interactions. This point is perhaps best seen with the less performing machine learning models, which can achieve the highest increases in accuracy levels just by using an appropriate “adaptive filter”. Although much research effort is spent in the advancement of machine learning models which can achieve high performance in discrimination tasks, we wish for this to be also a push beyond just the right choice of classifier, but rather into the right choice of physical system, which may *enable* previously unhelpful models. Although the improvements shown in these experiments are encouraging, further experiments in this direction are necessary to show the significance of these improvements under a varied range of experimental settings.

The method proposed is widely applicable on different object surfaces, where adaptation is induced by means of simple soft deformation. The methodology utilized to compare the performance of each filter, however, is limited by the ability in real-life scenarios to retrieve separate tactile sensor data for each type of filter in turn. For a large set of filter morphologies, this can be an untimely experimental process. The limitation on spatial resolution is another consideration. There is a fundamental limitation of the minimal spatial resolution that can be achieved with this approach, which is dependent on the thickness of the material covering the jamming material, and the size of the jamming particles. Moreover, there is bottle neck due to the sensor’s spacial resolution which is approximately 3 mm.

Whilst this ability to change the sensor structure shows promise, there is much scope to explore methods of optimizing the structure of the sensor online. Utilizing 3D printing could allow for custom templates to be printed in minutes to enable custom filter shapes to be generated rapidly. This could allow either optimization of the sensor structure using evolutionary experiments, or utilize simulation and modelling to predict the optimal sensory structure for a given task. Exploring this direction further could allow movement towards having a sensor that can be optimized for any, unseen sensory task.

## Data Availability

The raw data supporting the conclusions of this article will be made available by the authors, without undue reservation.
